# Familial aggregation and socio-demographic correlates of taste preferences in European children

**DOI:** 10.1186/s40795-017-0206-7

**Published:** 2017-12-06

**Authors:** Hannah S. Jilani, Timm Intemann, Leonie H. Bogl, Gabriele Eiben, Dénes Molnar, Luis A. Moreno, Valeria Pala, Paola Russo, Alfonso Siani, Antonia Solea, Toomas Veidebaum, Wolfgang Ahrens, Antje Hebestreit, on behalf of the I.Family consortium

**Affiliations:** 1 Leibniz Institute for Prevention Research and Epidemiology - BIPS, Bremen, Germany; 2 Institute of Statistics, Faculty of Mathematics and Computer Science, University of Bremen, Bremen, Germany; 3 Institute for Molecular Medicine FIMM, Helsinki, Finland; 4 Department of Public Health and Community Medicine, University of Gothenburg, Gothenburg, Sweden; 5 Department of Pediatrics, University of Pécs, Pécs, Hungary; 6 GENUD (Growth, Exercise, Nutrition and Development) Research Group, Faculty of Health Sciences, University of Zaragoza, Zaragoza, Spain; 7 Department of Preventive and Predictive Medicine, Fondazione IRCCS Istituto Nazionale dei Tumori, Milan, Italy; 8 Institute of Food Sciences, National Research Council, Avellino, Italy; 9 Research and Education Institute of Child Health, Strovolos, Cyprus; 10 Department of Chronic Diseases, National Institute for Health Development, Tallinn, Estonia

**Keywords:** Familial aggregation, Taste preferences, Children, Cross-cultural, Europe

## Abstract

**Background:**

Studies on aggregation of taste preferences among children and their siblings as well as their parents are scarce. We investigated the familial aggregation of taste preferences as well as the effect of sex, age, country of residence and education on variation in taste preferences in the pan- European I.Family cohort.

**Method:**

Thirteen thousand one hundred sixty-five participants from 7 European countries, comprising 2,230 boys <12 years, 2,110 girls <12 years, 1,682 boys ≥12 years, 1,744 girls ≥12 years and 5,388 parents, completed a Food and Beverage Preference Questionnaire containing 63 food items representing the taste modalities sweet, bitter, salty and fatty. We identified food items that represent the different taste qualities using factor analysis. On the basis of preference ratings for these food and drink items, a preference score for each taste was calculated for children and parents individually. Sibling and parent-child correlations for taste preference scores were calculated. The proportion of variance in children’s preference scores that could be explained by their parents’ preference scores and potential correlates including sex, age and parental educational was explored.

**Results:**

Mean taste preferences for sweet, salty and fatty decreased and for bitter increased with age. Taste preference scores correlated stronger between siblings than between children and parents. Children’s salty preference scores could be better explained by country than by family members. Children’s fatty preference scores could be better explained by family members than by country. Age explained 17% of the variance in sweet and 16% of the variance in fatty taste preference. Sex and education were not associated with taste preference scores.

**Conclusion:**

Taste preferences are correlated between siblings. Country could explain part of the variance of salty preference scores in children which points to a cultural influence on salt preference. Further, age also explained a relevant proportion of variance in sweet and fatty preference scores.

**Electronic supplementary material:**

The online version of this article (10.1186/s40795-017-0206-7) contains supplementary material, which is available to authorized users.

## Background

Taste preferences are the main food choice driver, especially in children for whom aspects such as healthiness and economics, e.g. food prices, generally play a minor role [1, 2].

If taste and food preferences derive from mere exposure, availability and familiarity, then the taste and food preferences of children should resemble that of their parents because they share meals and parents influence the availability of foods and drinks in the home [3, 4]. Further, it can also be assumed that the shared genetic information, environmental factors, as well as close personal interaction lead to similar taste and food preferences.

Previous studies from the 1980s however only observed weak positive correlations between taste and food preferences of children and their parents [5–9]. No differences were observed between the correlation of mothers and fathers with preferences of either boys or girls. The largest correlations were observed between spouses and between siblings as described in a review by Rozin [9]. Rozin then argued that the observed relationship between taste and food preferences should have been stronger for parents and their children due to the close relationship and the shared genetic characteristics (family paradox) [9]. Birch on the other hand concluded that weak correlations within families occurred due to communalities of a cultural group and underlined the need for cross-cultural research in this field [6]. Studies reported in a review by Reed et al. analysing the heritability of fat preference measured through fat intake in family and twin studies described a narrow sense heritability between 0 and 0.48 [10]. Previous studies in twin children based on questionnaires show a moderate genetic basis for food preferences in children [11, 12] and adolescence [13]. The shared family environment influences food preference of young children, but this influence disappears already in adolescence [13] and is also absent in adults [14]. Studies in Finnish adult twins that evaluated taste preferences by taste tests confirm a moderate heritability for individual differences in sweet taste preferences (41% for the strongest sucrose solution) [15] and further show that 34-50% of the variation in pleasantness of sour foods [16] and 18-58% of the variation in the pleasantness of oral pungency and spicy foods [17] can be attributed to genetic factors.

Preferences for sweet and fatty as well as aversion to bitter are innate [18, 19] and change during childhood. The age of the child can therefore also influence taste preferences. Further, children’s diet is associated with their parents’ educational level [20], presenting another possible influencing factor on children’s taste preferences.

Taste and food preferences develop during childhood and the process may persist until later in life [21]. Therefore, it is of great importance to understand how these preferences develop and how they can be influenced to support healthy food choices. Thus it is of interest to study the hypothesis that the taste preference of children resembles that of their parents.

The aim of this study was to assess food preferences of children and their parents, to identify foods representing the sweet, salty, fatty and bitter taste, and to investigate the association between sweet, salty, fatty and bitter taste preferences of children from different age-groups, their siblings and their parents from seven European countries. Further, the effect of sex, age, parental education and country of residence on taste preferences was investigated.

## Methods

### Study group

I.Family is a European multi-centre longitudinal study that presents the follow-up of the IDEFICS (**I**dentification and prevention of Dietary- and lifestyle-induced health EFects In Children and infantS) cohort [22, 23]. Between March 2013 and April 2014, all children that participated in the IDEFICS study were invited to take part in I.Family. Additionally, their siblings and parents were invited to the follow-up examinations. For the taste preference analysis, we included all participants from the age of 6 years onwards. From this sub-group, 13,165 participants (2,230 boys <12 years (also referred to as younger boys), 2,110 girls <12 years (also referred to as younger girls), 1,682 boys ≥12 years (also referred to as older boys), 1,744 girls ≥12 years (also referred to as older girls) and 5,399 parents) who fulfilled the inclusion criteria (age, sex, measured height, weight and biological relationship) completed the Food and Beverage Preference Questionnaire. Our study group comprises 5,128 child-mother dyads (with 3,588 mothers) and 3,223 child-father dyads (with 1,811 fathers) from 7 European countries (Cyprus, Estonia, Germany, Italy, Hungary, Spain and Sweden).

The large sample size of the I.Family study allowed conducting age-group specific analyses. Therefore, for the analysis the children were divided in boys <12 years, girls <12 years, boys ≥12 years, girls ≥12 years. The cut-off of 12 years was chosen because children 12 years and older are entering adolescence and therefore other factors like peers and growing independency might influence taste preferences whereas smaller children are more dependent on their parents with regard to food availability. The cut-off of 12 years seems reasonable not only for these social aspects but also for biological aspects. In a sub-sample of children (n=7123 children) information on breaking of the voice (for boys) and onset of menarche (for girls) was available. According to these characteristics a proportion of 84% of children classified as pubertal were ≥12 years old and 11% of children classified as pubertal were <12 years. In an even smaller sub-sample (n=5286) information for Tanner stages according to pubic hair (for boys) and breast development (for girls) was available. According to these characteristics 97% of prepubertal children were <12 years and 95% of pubertal children were ≥12 years old.

Each study centre obtained ethical approval from its local responsible institutional review board. Parents gave written informed consent for themselves and for their children. Adolescents 12 years and older gave their own written informed consent. All children were informed orally and gave their oral consent to participate in our study.

### Questionnaire and anthropometric measurements

We obtained information on sex, age and highest level of education for each participant using self-completion questionnaires. Parents completed their own questionnaire as well as for their children under twelve years old. Adolescents twelve years and older completed the questionnaire on their own. For each parent we categorised the highest educational level acquired according to the International Standard Classification of Education (ISCED) ranging from 1 (low education) to 8 (high education) [24]. For the present analysis the education level was grouped into three categories; ‘low education’ (ISCED level 0-2), ‘medium education’ (ISCED level 3-5) and ‘high education’ (ISCED level 6-8).

The height and weight of all participants were measured in a fasting state. The body mass index (BMI) was calculated for all participants and for all children it was converted into age- and sex-specific z-scores [25]. Participants were classified as thin/normal weight and overweight/obese (weight status) using age- and sex-specific cut-points published [25] for children. For adults, the cut off of 25 kg/m^2^ was chosen to classify parents as overweight/obese [26].

### Food and Beverage Preference Questionnaire

We developed a questionnaire that assessed preferences for sweet, salty, fatty and bitter and could be applied in children/adolescents as well as in adults. Duffy et al. described a preference questionnaire as useful for epidemiological studies to connect chemosensation with health outcomes [27]. Previously, a preference questionnaire for French adults was tested for reliability and collected data showed associations between assessed preferences and health outcomes as well as dietary intake [28–30].

**Fig. 1:**
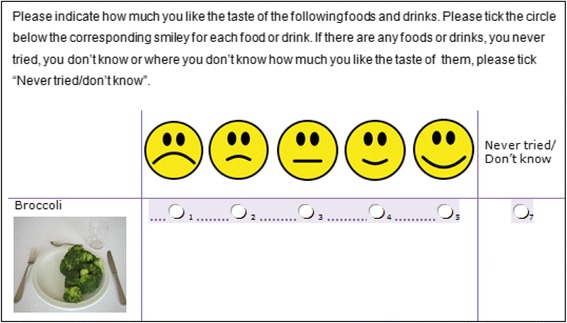
Example (screen shot) from the food and beverage preference questionnaire

We mainly compiled foods and drinks that were included in earlier food and beverage preference questionnaires [28, 31]. The questionnaire contained food photographs that were appropriate to be used in all age groups (Figure 1). In total, the questionnaire consisted of 63 items including single foods (e.g. banana, spinach), mixed foods (e.g. hot dog, kebab), condiments (e.g. jam, mayonnaise) and drinks (e.g. coke, lemonade).

Participants were asked to indicate how much they liked the taste of the food presented on the pictures using a 5 point likert (smiley-)scale, ranging from disliking to liking. Thus the variable of liking for each food and drink item ranged from 1 to 5, with 1 meaning ‘do not like at all’ and 5 meaning ‘like very much’. Additionally, participants could indicate that they do not know or have never tasted the specific food item. A pre-test was conducted in every country to ensure the feasibility of all food items across countries.

### Sensory taste preference score

Only foods that were ranked by at least 75% of the participants were included in this analysis. Participants were excluded when they had more than 20 missing or “Never tried/ Don’t know” answers. To assess the associations between foods and beverages, a latent variable exploratory factor analysis was conducted [32]. Further, a sex and age specific factor analysis was conducted to gain more accurate information about the factorial structure of food preference. The strata were boys <12 years, girls <12 years, boys ≥12 years, girls ≥12 years, and their mothers and fathers. We used the oblimin transformation, which allowed an analysis using non-orthogonal factors [33]. Different diagnostic tools were applied to identify an appropriate number of factors including Horn’s parallel test, Wayne Velicer's Minimum Average Partial criterion and the optimal coordinates index [34]. We chose a 13 factor solution for every age and sex specific group. A food or drink item was considered to belong to a particular factor if the factor loading was greater than 0.30 on that factor. The factor analysis explained between 32% and 41% of the overall variance in the variables (fathers 41%, mothers 39%, older girls 36%, older boys 38%, younger boys 37% and younger girls 32%). We then used the obtained factors to conduct a content analysis in order to assign the factors to the taste modalities sweet, salty, fatty and bitter (Table 1). Food and drink items with no load on one of the factors were not included in further analyses.

**Table 1:** Foods and drinks representing four taste modalities

	Boys <12 years	Girls <12 years	Boys ≥12 years	Girls ≥12 years	Fathers	Mothers
Sweet						
Milk chocolate	X	X	X	X	X	X
Chocolate bar	X	X	X	X	X	X
Lemonade	X	X		X	X	X
Coke	X	X	X	X	X	X
Diet coke	X		X	X	X	X
Donut	X		X	X	X	X
Jam	X	X	X	X		X
Honey	X	X	X	X	X	X
Plain croissant		X	X	X	X	X
Chocolate croissant	X	X		X	X	X
Cornflakes	X	X	X	X	X	X
Chocolate crispies		X	X	X	X	X
Chocolate spread	X	X	X	X	X	X
Banana	X	X		X	X	
Fruit yoghurt	X	X	X	X	X	X
Yoghurt	X		X	X	X	X
Fruit juice	X	X	X	X	X	X
Chocolate pudding		X	X			
Gateau			X		X	X
Ice tea					X	
Ice cream					X	X
Water					X	
Wholemeal bread					X	
Salty						
Salt			X			
Salted nuts	X	X	X	X	X	X
Salted pistachios	X	X	X	X	X	X
Savoury biscuits	X	X	X	X	X	X
Salty sticks	X	x	X	X	X	X
Olives					X	
Feta					X	
Fatty						
Hamburger	X	X	X	X	X	X
Hot Dog	X	X	X	X	X	X
Fried chicken	X		X	X	X	X
Steak	X			X	X	
French fries	X	X	X	X		X
Chips	X	X	X	X		X
Sausage	X	X	X	X	X	X
Salami	X			X	X	X
Butter	X	X	X	X	X	X
Mayonnaise	X		X	X	X	X
Milk		X		X	X	
Cream			X	X	X	X
Mashed potatoes			X			
Kebab				X	X	X
Nachos				X		X
Chili sauce				X		X
Bitter						
Broccoli	X	X	X	X	X	X
Spinach	X	X	X	X	X	X
Lettuce	X		X			
Olives	X		X			X
Lasagne			X			
Red cabbage					X	X
Sprouts					X	X
Asparagus					X	X
Grapefruit						X
Steak						X

Foods that did not load on any factor: For boys <12 years: whole meal bread, lasagne, cream, whole milk skimmed milk, mashed potatoes, sausage, broth, salt, nachos, choco crispies, wine gum, dark chocolate, water, donut, ice cream, ice tea, plain croissant, cream gateau. For girls <12years: whole meal bread, lasagne, lettuce, cream, mayonnaise, mashed potatoes, fried chicken, steak, broth, olives, salami, salt, nachos, wine gum, dark chocolate, yoghurt, water, donut, ice cream, ice tea. For older boys ≥12 years: whole meal bread, chili, grape fruit, whole milk skimmed milk, steak, broth, salami, kebab, nachos, banana, lemonade, wine gum, dark chocolate, water. For older girls ≥12 years: whole meal bread, lasagne, lettuce, grape fruit, skimmed milk, mashed potatoes, broth, olives, salt, wine gum, dark chocolate water, chocolate pudding, ice cream, ice tea, cream gateau. For fathers: whole meal bread, coffee, lasagne, chili, lettuce, beet, grape fruit, skimmed milk, mashed potatoes, avocado, broth, french fries, Crisps, nachos, wine gum, dark chocolate, chocolate pudding, jam. For mothers: whole meal bread, coffee, lasagne, lettuce, beer, whole milk, skimmed milk, avocado, broth, feta, salt, banana, wine gum, dark chocolate, water, chocolate pudding, ice tea.

We computed scores for liking of the specific taste modality by calculating the mean liking of the foods and drinks included in each of the 4 categories. Scores were calculated individually for younger boys, younger girls, older boys, older girls well as their mothers and fathers. To this end we calculated the sum of the ratings for the foods and drinks and divided the sum by the number of foods and drinks that were included in the specific taste modality group.

### Statistical analysis

Descriptive analysis of study characteristics of the study population were conducted by each stratum (boys <12 years, girls <12 years, boys ≥12 years, girls ≥12 years their mothers and fathers) as well as by each participating country. We also calculated the quartiles (median, p25, p75) of sweet, salty, fatty and bitter liking scores of each stratum.

To adjust for the effect of age on taste preferences, age standardised residuals from taste preference scores were obtained from regression analyses separately for each stratum. The residuals were used to analyse the associations between taste preferences of parents and children as well as between and among younger and older siblings. We estimated inter- and intraclass correlations for all relative pairs of a family using the FCOR (family correlations) program in SAGE (Statistical Analysis for Genetic Epidemiology software), version 6.3 [35].

In a sub-group analysis we analysed the correlations of taste preferences between parents and their children as well as between siblings separately for those children whose father and mother had similar preferences (difference between mother’s and father’s preference score between -1 and 1) vs. those children whose father and mother had different preferences (difference between mother’s and father’s preference score below -1 or above 1). Rozin supposed that children from parents with incongruent preferences might receive a ‘mixed message’, which might lead to a disappearance of the familial aggregation effect [9].

Additionally, for each sex-by-age stratum, we estimated the proportion of variance in sweet, salty, fatty and bitter preference scores that could be explained by mother’s, father’s, brothers’ and sisters’ preference scores and country (potential correlates). We estimated several linear mixed models: a null model, including only a random intercept term for family membership and another model, including the random intercept term and each of the potential correlate only. Based on these models we calculated the proportion of variance in children’s taste preference scores that could be explained by preference scores of mothers, fathers, brothers and sisters. Additionally, we calculated the proportion of variance in taste preference scores that could be explained by country. To assess the impact of sex, age and highest education level on taste preferences, we used non-stratified taste preference scores (all children and parents) for each taste modality as dependent variables in a linear mixed model. Sample sizes for these analyses varied due to missing values for particular covariates (e.g. parent or sibling information).

The factor analysis was conducted using statistical software R, version 3.1.0 [36]. Familial correlations were conducted using SAGE. All other analyses were carried out using the statistical software SAS (Statistical Analysis System, SAS Institute Inc., Cary, USA), version 9.3.

## Results

### Study characteristics

Thirteen thousand one hundred sixty-five participants from 7 European countries, comprising 7,766 children and 5,399 parents participated in our study. 49.6% of the children and 66.5% of the parents were female. 28.1% of the children and 56.6% (48% of mothers and 74% of fathers) of the parents were overweight or obese and 53.6% of the families had at least one parent with a high education. More detailed characteristics can be found in Table 2. Country-specific characteristics can be found as Additional file 1: Table S1.

**Table 2:** Characteristics of the study sample

	Mothers	Fathers	Boys <12y	Girls <12y	Boys ≥12y	Girls ≥12y
	*N* = 3588	*N* = 1811	*N* = 2230	*N* = 2110	*N* = 1682	*N* = 1744
	Mean (SD)	Mean (SD)	Mean (SD)	Mean (SD)	Mean (SD)	Mean (SD)
Age	41.4 (5.3)	44.5 (5.9)	9.6 (1.5)	9.7 (1.5)	13.6 (1.1)	13.6 (1.1)
BMI	25.8 (5.3)	27.9 (4.4)	18.3 (3.7)	18.3 (3.6)	21.0 (4.3)	21.2 (4.2)
BMI z-score^a^	-	-	0.6 (1.2)	0.5 (1.1)	0.7 (1.2)	0.6 (1.1)
	N (%)	N (%)	N (%)	N (%)	N (%)	N (%)
Overweight/obese	1698 (47.9)	1316 (74.0)	616 (27.8)	596 (28.3)	505 (30.1)	457 (26.3)
Low education^b^	162 (4.7)	43 (2.5)	-	-	-	-
Medium education^b^	1540 (44.3)	670 (38.6)	-	-	-	-
High education^b^	1772 (51.0)	1023 (58.9)	-	-	-	-
Italy	591 (16.5)	179 (9.9)	400 (17.9)	352 (16.7)	323 (19.2)	321 (18.4)
Estonia	520 (14.5)	224 (12.3)	311 (14.0)	288 (13.7)	227 (13.5)	275 (15.8)
Cyprus	726 (20.2)	466 (25.7)	529 (23.7)	518 (24.6)	413 (24.6)	393 (22.5)
Sweden	335 (9.3)	163 (9.0)	242 (10.9)	215 (10.2)	143 (8.5)	147 (8.4)
Germany	561 (15.6)	247 (13.6)	311 (14.0)	293 (13.9)	262 (15.6)	281 (16.1)
Hungary	632 (17.6)	377 (20.8)	268 (12.0)	277 (13.1)	230 (13.7)	227 (13.0)
Spain	223 (6.2)	155 (8.6)	169 (7.6)	167 (7.9)	84 (5.0)	100 (5.7)

*Abbreviations*: *y* year, *BMI* Body mass index

^a^:BMI z-scores according to Cole and Lobstein 2012 [25]

^b^:International Standard Classification of Education Maximum (ISCED); maximum of both parents (0, 1, 2 = low education; 3, 4 = medium education; 5, 6 = high education) [24]

The median (p25;p75) family size was 3.0 (2.0;4.0), ranging from 1 to 7. Numbers of different family types can be found in Table 3. The most abundant family type was a mother with 1 child (24.4%). But also mother and father with 1 or 2 children represented together 23.8%.

**Table 3:** Numbers of family types of the study sample

Family types	No. of families	%
Mother only	57	1.0
Mother – 1 child	1343	24.4
Mother – 2 children	632	11.5
Mother – 3 children	83	1.5
Mother – 4 children	14	0.3
Mother – 5 children	1	0.0
Fathers only	23	0.4
Father – 1 child	203	3.7
Father – 2 children	112	2.0
Father – 3 children	15	0.3
Father – 4 children	2	0.0
Father and Mother only	21	0.4
Father and Mother – 1 child	694	12.6
Father and Mother – 2 children	618	11.2
Father and Mother – 3 children	110	2.0
Father and Mother – 4 children	10	0.2
Father and Mother – 5 children	2	0.0
1 child only	1143	20.7
2 children	380	6.9
3 children	41	0.7
4 children	8	0.1
5 children	1	0.0
Total	5513	100

Excluding food items which were known to or tasted by less than 75% of the participants led to the following exclusions in the stratum of younger boys: Asparagus, brussels sprouts, beer, black coffee, chili sauce, grapefruit, red cabbage, avocado, feta and kebab. In the stratum of younger girls: Asparagus, brussels sprouts, beer, black coffee, chili sauce, grapefruit, red cabbage, diet coke, avocado, feta and kebab. In the stratum of older boys: Asparagus, brussels sprouts, beer, black coffee, red cabbage, avocado and feta. In the stratum of older girls: Asparagus, brussels sprouts, beer, black coffee, red cabbage, avocado and feta. In the groups of mothers and fathers no food or drink items were excluded.

Quartiles (median, p25, p75) of the subsequently calculated scores are displayed in Table 4. Highest scores were achieved for the sweet and fatty score in young children. Lowest scores were observed in children for the bitter score. Parents had higher bitter scores than children.

**Table 4:** Age and sex specific distribution of sweet, salty, fatty and bitter taste preference scores (median, p25, p75)

	Boys (< 12 years)	Girls (< 12 years)	Boys (≥12)	Girls (≥12)	Fathers	Mothers
Sweet score						
N	2227	2106	1681	1743	1810	3587
Median(p25;p75)	4.2 (3.8;4.6)	4.2 (3.7;4.6)	4.1 (3.7;4.5)	4.1 (3.6;4.4)	3.6 (3.1;4.0)	3.5 (3.0;3.9)
Salty score						
N	2175	2073	1663	1734	1803	3568
Median(p25;p75)	4.0 (3.3;4.8)	4.0 (3.3;4.8)	3.8 (3.0;4.3)	3.8 (3.0;4.5)	3.7 (3.0;4.2)	3.5 (2.8;4.0)
Fatty score						
N	2228	2109	1682	1743	1809	3587
Median(p25;p75)	4.3 (3.8;4.6)	4.3 (3.7;4.6)	4.2 (3.8;4.5)	3.9 (3.5;4.3)	3.7 (3.3;4.2)	3.4 (2.9;3.9)
Bitter score						
N	2197	1939	1677	1658	1809	3587
Median(p25;p75)	3.0 (2.3;4.0)	3.0 (2.0;4.0)	3.4 (2.8;4.0)	3.0 (2.0;4.0)	3.6 (3.0;4.2)	3.8 (3.3;4.3)

### Correlations of taste preferences among family members

Table 5 shows the results for interclass correlations between children from different age and sex strata as well as mothers and fathers and intraclass correlations among siblings for residuals of sweet, bitter, salty and fatty scores.

**Table 5:** Familial correlations (r), standard errors (SE) of the mean and p-values for residuals of sweet, salty, fatty and bitter taste preference scores

	Relationship		Sweet	Salty	Fatty	Bitter
Parent-offspring		No. of pairs	7838	7623	7849	7451
		r ± SE	0.16 ± 0.01	0.08 ± 0.01	0.15 ± 0.01	0.14 ± 0.01
		*p*-value	<0.0001	<0.0001	<0.0001	<0.0001
Parent-offspring	<12 years	No. of pairs	4501	4358	4500	4215
		r ± SE	0.14 ± 0.02	0.08 ± 0.02	0.15 ± 0.02	0.13 ± 0.02
		*p*-value	<0.0001	<0.0001	<0.0001	<0.0001
	≥12 years	No. of pairs	3337	3265	3349	3236
		r ± SE	0.18 ± 0.02	0.09 ± 0.02	0.15 ± 0.02	0.14 ± 0.02
		*p*-value	<0.0001	<0.0001	<0.0001	<0.0001
Mother-daughter	<12 years	No. of pairs	1453	1411	1453	1336
		r ± SE	0.14 ± 0.03	0.10 ± 0.03	0.18 ± 0.03	0.18 ± 0.03
		*p*-value	<0.0001	0.0003	<0.0001	<0.0001
	≥12 years	No. of pairs	1085	1064	1091	1039
		r ± SE	0.19 ± 0.03	0.09 ± 0.03	0.19 ± 0.03	0.12 ± 0.03
		*p*-value	<0.0001	0.0026	<0.0001	0.0002
Mother-son	<12 years	No. of pairs	1486	1433	1487	1434
		r ± SE	0.16 ± 0.03	0.08 ± 0.03	0.13 ± 0.03	0.10 ± 0.03
		*p*-value	<0.0001	0.0029	<0.0001	0.0001
	≥12 years	No. of pairs	1095	1074	1097	1085
		r ± SE	0.19 ± 0.03	0.11 ± 0.03	0.11 ± 0.03	0.19 ± 0.03
		*p*-value	<0.0001	0.0007	0.0002	<0.0001
Father-daughter	<12 years	No. of pairs	755	735	753	680
		r ± SE	0.10 ± 0.04	0.05 ± 0.04	0.14 ± 0.04	0.18 ± 0.04
		*p*-value	0.0056	0.1781	0.0001	<0.0001
	≥12 years	No. of pairs	584	571	585	548
		r ± SE	0.20 ± 0.04	0.09 ± 0.40	0.10 ± 0.04	0.16 ± 0.04
		*p*-value	<0.0001	0.0269	0.0147	0.0002
Father-son	<12 years	No. of pairs	807	779	807	765
		r ± SE	0.15 ± 0.04	0.01 ± 0.04	0.15 ± 0.04	0.07 ± 0.04
		*p*-value	<0.0001	0.7291	0.0001	0.0527
	≥12 years	No. of pairs	573	556	576	564
		r ± SE	0.15 ± 0.04	0.06 ± 0.04	0.20 ± 0.04	0.10 ± 0.04
		*p*-value	0.0003	0.1851	0.0002	0.0258
Sibling-sibling	<12 years	No. of pairs	965	922	967	848
		r ± SE	0.26 ± 0.03	0.23 ± 0.03	0.26 ± 0.03	0.20 ± 0.03
		*p*-value	<0.0001	<0.0001	<0.0001	<0.0001
	≥12 years	No. of pairs	434	417	434	401
		r ± SE	0.15 ± 0.05	0.10 ± 0.05	0.08 ± 0.05	0.18 ± 0.05
		*p*-value	0.0026	0.0399	0.0953	0.0006
Brother-brother	<12 years	No. of pairs	287	274	287	264
		r ± SE	0.34 ± 0.05	0.20 ± 0.06	0.32 ± 0.06	0.35 ± 0.06
		*p*-value	<0.0001	0.0009	<0.0001	<0.0001
	≥12 years	No. of pairs	123	120	123	121
		r ± SE	0.17 ± 0.09	0.10 ± 0.09	0.15 ± 0.09	-0.01 ± 0.09
		*p*-value	0.0584	0.2824	0.1004	0.9035
	<12 years-≥12 years	No. of pairs	264	255	265	261
		r ± SE	0.34 ± 0.05	0.18 ± 0.06	0.30 ± 0.06	0.16 ± 0.06
		*p*-value	<0.0001	0.0035	<0.0001	0.0074
Sister-sister	<12 years	No. of pairs	237	225	238	195
		r ± SE	0.24 ± 0.06	0.32 ± 0.06	0.26 ± 0.06	0.21 ± 0.07
		*p*-value	0.0002	<0.0001	0.0001	0.0028
	≥12 years	No. of pairs	107	104	107	95
		r ± SE	0.24 ± 0.09	0.22 ± 0.10	0.09 ± 0.10	0.37 ± 0.09
		*p*-value	0.0128	0.0250	0.3802	0.0003
	<12 years-≥12 years	No. of pairs	248	244	248	226
		r ± SE	0.25 ± 0.06	0.15 ± 0.06	0.18 ± 0.06	0.26 ± 0.06
		*p*-value	<0.0001	0.0172	0.0028	0.0001
Sister-brother	<12 years	No. of pairs	441	423	442	389
		r ± SE	0.20 ± 0.05	0.20 ± 0.05	0.22 ± 0.05	0.11 ± 0.05
		*p*-value	<0.0001	<0.0001	<0.0001	0.0336
	≥12 years	No. of pairs	204	193	204	185
		r ± SE	0.09 ± 0.07	0.04 ± 0.07	0.03 ± 0.07	0.12 ± 0.07
		*p*-value	0.2121	0.6198	0.6526	0.1084
	<12 years-≥12 years	No. of pairs	233	226	233	212
		r ± SE	0.16 ± 0.06	0.21 ± 0.06	0.22 ± 0.06	0.19 ± 0.07
		*p*-value	0.0125	0.0012	0.0007	0.0048
	≥12 years-<12 years	No. of pairs	255	255	255	244
		r ± SE	0.25 ± 0.06	0.15 ± 0.06	0.17 ± 0.06	0.21 ± 0.06
		*p*-value	<0.0001	0.0155	0.0058	0.0011

Correlations showed significant but weak family aggregation for almost all taste modalities and types of relative pairs.

Among all types of parent-offspring pairs, correlations were highest (r=0.20) between fathers and daughters ≥12 years old for sweet and between fathers and sons ≥12 years old for fatty.

Sibling-sibling correlations (independent of sex) were highest (0.20 to 0.26) among siblings <12 years of age for all taste modalities, while those among ≥12 year old siblings ranged from 0.08 to 0.15. Brother-brother correlations ranged from -0.01 to 0.35 and were significant only for those <12 years of age for all taste modalities. Correlations among ≥12 year old brothers were not significant. Correlations between <12 year old brothers and ≥12 year old brothers ranged from 0.18 to 0.34 and were significant for sweet and fatty.

Sister-sister correlations for sisters <12 years of age ranged from 0.21 to 0.32 and were significant for all taste modalities. Correlations among ≥12 year old sisters ranged from 0.09 to 0.37 and were significant for sweet, salty and bitter. Correlations between <12 year old sisters and ≥12 year old sisters ranged from 0.15 to 0.26 and were significant for sweet and bitter.

Brother-sister correlations for brothers and sisters <12 years of age ranged from 0.11 to 0.22 and were significant for sweet, salty and fatty. Correlations between ≥12 year old brothers and sisters ranged from 0.03 to 0.12 and were not significant. Correlations between sisters <12 years of age and brothers ≥12 years of age ranged from 0.16 to 0.22 and were significant for salty and fatty. Correlations between brothers <12 years of age and sisters ≥12 years of age ranged from 0.15 to 0.25 and were significant for sweet and bitter.

The comparison of taste preferences between children whose parents both had similar preferences and those whose parents had different preferences showed that taste preferences of children from parents with same preferences correlated stronger to their parents’ preferences than those of children from parents with incongruent taste preferences (data not shown). The highest correlations were seen for sweet preference scores between <12 year old girls with ≥12 year old sisters (*r*=0.44) and between mothers and sons ≥12 years old (*r*=0.27) as well as for fatty preference scores between mothers and sons ≥12years old (r=0.33) and between fathers and sons ≥12 years old (r=0.34).

### Explanation of variance

Table 6 shows the proportion of variance in children’s taste preference scores that could be explained by their mother’s, father’s, brothers’ and sisters’ taste preference and country. The proportion of variance that could be explained by parents was highest for fat preference (between 4.3% and 8.3%). For girls and boys ≥12 years of age, 6.4% and 5.8%, respectively, of sweet taste preference score could be explained by their parents’ sweet taste preference score. The bitter taste preference score of <12 year old girls 6% of variance could be explained by parents’ bitter taste preference score. The proportions of variance in children’s taste preference score that could be explained by country were under 4% for all age and sex strata except for salt taste preference scores, where proportions of explained variance by country were between 5.4% and 7.5% for all age and sex strata.

**Table 6:** Percentage of children’s variance in preference scores that could be explained by preference scores of their family members and country

		% Variance by mother	% Variance by father	% Variance by parents	% Variance by male siblings <12 y	% Variance by female siblings <12 y	% Variance by male siblings ≥12 y	% Variance by female siblings ≥12 y	% Variance by country
Sweet score	Boys <12 y.	2.7	2.4	4.1	-	4.2	8.2	4.1	1.3
	Girls <12 y.	2.2	1.2	2.2	4.2	-	1.5	7.0	3.7
	Boys ≥12 y.	3.5	2.9	5.8	8.3	1.5	-	0.5	3.1
	Girls ≥12 y.	4.4	3.4	6.4	4.2	6.6	0.6	-	3.0
Salty score	Boys <12 y.	0.6	0^a^	1.0	-	4.3	3.7	1.2	5.4
	Girls <12 y.	1.1	0.6	0.7	4.4	-	4.9	1.3	7.5
	Boys ≥12 y.	1.4	0.4	1.9	3.9	4.1	-	0.2	5.8
	Girls ≥12 y.	1.0	0.6	1.3	1.2	1.3	0.3	-	6.4
Fatty score	Boys <12 y.	2.3	2.4	4.3	-	5.2	5.1	1.9	2.2
	Girls <12 y.	3.4	2.1	5.5	5.1	-	2.8	1.7	1.4
	Boys ≥12 y.	1.7	5.1	8.3	5.1	2.7	-	0^a^	3.8
	Girls ≥12 y.	3.8	0.8	6.1	1.9	1.6	0^a^	-	1.5
Bitter score	Boys <12 y.	0.7	0.2	0.7	-	0.7	4.0	4.1	2.7
	Girls <12 y.	3.1	2.8	6.0	0.9	-	3.4	8.5	2.4
	Boys ≥12 y.	3.0	1.1	2.2	4.1	3.4	-	0.8	1.6
	Girls ≥12 y.	1.3	2.9	3.1	4.8	7.9	0.9	-	2.7

^a^Due to model misspecification or lack of power negative variance percentage were estimated and therefore set to zero

Table 7 shows the proportions of variance in non-stratified taste preference scores that could be explained by sex, age and highest education level. Age explained 17%, 16% and 7% of sweet, fat and bitter preference, respectively. All other proportions of explained variance by sex and highest education level were below 5%.

**Table 7:** Percentage of variance in preference scores that could be explained by sex, age and highest education

	% Variance sweet core (n)	% Variance salty score (n)	% Variance fatty score (n)	% Variance bitter score (n)
Sex	1.2 (13,173)	0.4 (13,035)	4.4 (13,177)	0.0 (12,886)
Age	17.4 (13,173)	3.4 (13,035)	16.3 (13,177)	7.2 (12,886)
ISCED^a^	0.0 (12,679)	0.0 (12,549)	0.4 (12,684)	0.0 (12,405)

^a^International Standard Classification of Education [24]

## Discussion

In our study we analysed sweet, salty, bitter and fatty taste preferences among European families. We observed a decrease with age in sweet, salty and fatty preference scores, while bitter taste preference scores increased with age. Further, taste preference scores correlated stronger among siblings than between children and their parents. For all taste modalities correlations were highest among younger siblings and among older siblings only present in girls. Nevertheless, these age- and sex-group specific correlations need to be interpreted with more caution since they were not as powered as the overall correlations. Furthermore, we observed that 17%, 16% and 7% of total variance in the non-stratified sweet, fatty and bitter taste preference scores, respectively, were explained by age. The strong age effect on taste preferences indicated by these results might be evolutionary meaningful, similar to the innate preference for sweet as well as fatty and the aversion for bitter [37]. Another explanation might be the matured taste perception of parents; children have about five times more taste buds, and their foliate papillae are larger and more abundant compared to those of adults. Nevertheless, this does not consequently lead to higher taste sensitivity, due to the fact that children’s innervation of taste papillae is not fully developed. The development of the taste apparatus carries on through childhood [38, 39]. These age-related differences could explain stronger correlations among siblings compared to correlations of parents’ taste preferences with those of their children.

Our data confirm earlier observations of a stronger correlation of food preferences between siblings than between children and their parents [8]. As an explanation for these findings, Pliner and Pelchat suggested that siblings share more genetic information than children share with their parents [8]. In contrast to parents and children, siblings share 25% of the dominant genetic effects. Siblings may share more similar experiences (e.g. school, peers) as compared to their parents as they are closer in age. Additionally, if gene expression is age dependent, gene expression of siblings closer in age should is expected to be more similar.

Another factor that influences children’s taste and food preference is food neophobia, the rejection of new and unknown foods [37]. This phenomenon is reported to decrease with increasing age from childhood to adulthood [40]. Our observations when looking at the number of food and drink items excluded because of missing values before conducting the factor analysis are in line with this. The number of excluded items decreased with increasing age suggesting that with increasing age the participants get familiar with a greater variety of foods and drinks. This was supported by our factor analysis that showed an increasing number of items per taste modality with increasing age of participants.

Beside these biological relationships, social factors may also account for our findings. As children grow older, their attitudes towards foods and drinks change [41] and the influences of peers become stronger [42]. This could be an explanation for the low correlation among older boys. Older female siblings in our study still resembled each other.

Furthermore, parental encouragement and family rules have been reported to affect the eating habits of children [43, 44]. Parents may tend to offer a healthier diet to younger children compared to adolescents. Especially mothers are more aware and adhere more to dietary guidelines also when feeding their children [45]. These facts may lead to different exposure for younger children than older children which may be another explanation for the stronger correlations among younger children compared to older children or children-parent correlations. Fathers in contrast have been found to have a high influence on a child’s sweet and fatty food choice, including all types of sugar, sweets, unhealthy drinks such as soft drinks and unhealthy fats [46]. This is in line with our results showing that in particular the sweet preference scores of fathers could partly explain their children’s sweet preference scores. Further, the correlations between fathers and daughters were observed to be high for sweet and between fathers and sons high in fat preference.

While our study has the strength that it includes data from more than 7,000 children and 5,000 parents from 7 European countries, some methodological aspects need to be addressed. Logue et al. stated 6 conditions that must be fulfilled to investigate familial aggregation in food preferences. *1. The range of examined foods must be ample enough and should not include only commonly liked or disliked foods. Additionally, the used scale must be wide enough.* In the present study we chose a wide variety of foods and drinks that produced a broad range of answers on likes and dislikes. It is however still possible that the number of food items that were chosen influenced the factorial structure that we obtained. A study conducted by Skinner et al. included 194 food items, whereas other studies included 59, 47, 32, 94 [5, 7, 9, 32, 47]. Since we included children as young as 6 years old, we needed a scale simple enough to be understood and answered also in that age range. According to the ‘ASTM Guide for sensory evaluation of products by children and minors’, six year old children are able to answer simple liking scales [48]. *2. The sample size should be large enough.* A main strength of our study is the large sample size including a large number of children, adolescents and parents. To our knowledge there is only one study that included more participants, but the study was conducted only in adults [28]. *3. Sex differences should be taken into account and 4. The preferences should be reported by each participant him/herself and no proxy should be used.* It has been discussed in the literature that parents’ reports about their children’s preferences in the context of comparing children’s and parents’ preferences might pull the answers in the direction of parents’ preferences [1, 5, 49]. In conformity with Logue et al. we stratified our analysis by sex and every participant completed the questionnaire by him/herself*. 5. The biological relationship between children and parents should be taken into account and lastly, the participating children should be living together with their parents.* We included only biological parents and assumed that they were living together with their children because they participated in the study as a family and children were rather young. Another strength of this study is the availability of other additional correlates of taste preferences such as parental educational and country of residence.

Using a food and beverage preference questionnaire to assess taste preferences seemed feasible in a large-scale epidemiological study. Asking for preferences for different foods and drinks with different tastes considers multiple sensory factors that have an influence on actual preferences which are relevant for real life, such as taste sensitivity, taste intensity, social factors, and environmental factors as claimed by Hayes and Keast [50].

## Conclusion

To our knowledge this is the first European multicentre epidemiological study investigating the familial aggregation of taste preferences in a high number of participants from seven European countries, following a standardized study design. We conclude that the family paradox stated by Rozin still remains partly unsolved [9]. The hypothesis that children resemble their parents’ food and taste preferences could only be partly confirmed. Nevertheless, we found a correlation of taste preferences among siblings. This finding does indicate that there are similarities among family members. Age could explain part of the variance in sweet and fatty preference scores. Country could explain part of the variance of salty preference scores in children which points to a cultural influence on salt preference. No other studied correlate was associated with taste preference scores.

## Additional file

Additional file 1: Table S1. Country specific characteristics of the full study sample. (DOCX 15 kb)

## Abbreviations

BMI: Body Mass Index
IDEFICS Study: Identification and prevention of Dietary- and lifestyle-induced health EFfects In Children and infantS Study
ISCED: International Standard Classification of Education
SAS: Statistical Analysis System
