# Supplementary material for: Familial aggregation and socio-demographic correlates of taste preferences in European children
Source: BMC Nutr. 2017 Dec 6;3:87. doi: 10.1186/s40795-017-0206-7 (PMC7050807; doi:10.1186/s40795-017-0206-7)
Supplement: Supplementary file 1 — Country specific characteristics of the full study sample. (DOCX 15 kb) [file 40795_2017_206_MOESM1_ESM.docx]

Additional file 1

Table S1: Country specific characteristics of the full study sample

|  | Italy  (n=2,166; 16.5%) | Estonia  (n=1,831; 13.9%) | Cyprus  (n=3,045; 23.2%) | Sweden  (n=1,248; 9.5%) | Germany  (n=1,955; 14.9%) | Hungary  (n=2,011; 15.3%) | Spain  (n=898; 6.8%) | Total  (n=13,154) |
| --- | --- | --- | --- | --- | --- | --- | --- | --- |
| **Children (n)** | **1,396** | **1,101** | **1,853** | **747** | **1,147** | **1,002** | **520** | **7,764** |
| Girls (%) | 48.2 | 51.1 | 49.2 | 48.5 | 50.0 | 50.3 | 51.4 | 49.6 |
| Age  (mean (SD)) | 11.3  (2.2) | 11.5  (2.4) | 11.3  (2.7) | 11.1  (2.2) | 11.7  (2.4) | 11.5  (2.2) | 10.9  (2.1) | 11.4  (2.4) |
| BMI (kg/m^2^)  (mean (SD)) | 21.6  (4.4) | 18.9  (3.6) | 19.8  (4.4) | 17.8  (2.8) | 19.1  (3.9) | 18.9  (4.2) | 18.5  (3.2) | 19.5  (4.1) |
| BMI z-score (mean (SD)) | 1.2  (1.1) | 0.4  (1.0) | 0.7  (1.2) | 0.1  (1.0) | 0.4  (1.1) | 0.3  (1.2) | 0.4  (1.0) | 0.6  (1.1) |
| Overweight/  Obese (%) | 50.4 | 19.3 | 32.0 | 12.1 | 22.6 | 21.3 | 20.7 | 28.1 |
| **Parents (n)** | **770** | **730** | **1,192** | **501** | **808** | **1,009** | **378** | **5,399** |
| Mothers (%) | 76.8 | 70.1 | 60.9 | 67.3 | 69.4 | 62.6 | 59.3 | 66.5 |
| Age  (mean (SD)) | 43.3  (5.6) | 40.1  (5.4) | 42.3  (6.0) | 43.9  (5.4) | 43.3  (6.2) | 41.4  (5.4) | 45.5  (4.4) | 42.5  (5.8) |
| BMI (kg/m^2^)  (mean (SD)) | 27.7  (5.1) | 25.8  (5.1) | 27.2  (5.3) | 24.7  (3.8) | 27.0  (5.3) | 26.6  (5.3) | 25.2  (4.2) | 26.6  (5.1) |
| Overweight/  obese (%) | 69.1 | 46.9 | 61.5 | 38.8 | 59.7 | 57.9 | 47.0 | 56.6 |
| Low education (%) | 14.6 | 0.0 | 1.4 | 0.0 | 6.5 | 2.5 | 1.6 | 4.0 |
| Medium education (%) | 63.0 | 24.8 | 38.5 | 22.8 | 54.8 | 48.8 | 30.7 | 42.4 |
| High education (%) | 22.4 | 75.2 | 60.2 | 77.2 | 38.7 | 48.8 | 67.7 | 53.6 |
